# Oculomotor Nerve Palsy—Etiologies, Symptoms and Diagnosis: A Systematic Review with Meta-Analysis

**DOI:** 10.3390/diagnostics16091401

**Published:** 2026-05-06

**Authors:** Konstantina Bolou, George Triantafyllou, Nikolaos-Achilleas Arkoudis, Panagiotis Papadopoulos-Manolarakis, Irini Chatziralli, Vasileios Papadopoulos, Georgios Velonakis, Maria Piagkou

**Affiliations:** 1 School of Medicine, Faculty of Health Sciences, National and Kapodistrian University of Athens, 11527 Athens, Greece; kmpolou@gmail.com; 2 Department of Anatomy, School of Medicine, Faculty of Health Sciences, National and Kapodistrian University of Athens, 11527 Athens, Greece; georgerose406@gmail.com (G.T.); p.papado89@gmail.com (P.P.-M.); 3 Research Unit of Radiology and Medical Imaging, National and Kapodistrian University of Athens, 11528 Athens, Greece; nick.arkoudis@gmail.com (N.-A.A.); gvelonakis@med.uoa.gr (G.V.); 4 Second Department of Radiology, General University Hospital “Attikon”, National and Kapodistrian University of Athens, 12462 Athens, Greece; 5 Department of Neurosurgery, General Hospital of Nikaia-Piraeus, 18454 Athens, Greece; 6 Second Department of Ophthalmology, General University Hospital “Attikon”, National and Kapodistrian University of Athens, 12462 Athens, Greece; eirchat@yahoo.gr; 7 Laboratory of Anatomy, Faculty of Medicine, Democritus University of Thrace, 69100 Alexandroupolis, Greece; vapapadop@med.duth.gr

**Keywords:** oculomotor nerve, oculomotor nerve palsy, paresis, neuro-ophthalmology, neurology, evidence-based medicine, meta-analysis

## Abstract

**Background/Objectives:** Oculomotor nerve palsy (OMNP) is a clinically significant condition that may represent the earliest manifestation of life-threatening intracranial pathology, particularly aneurysmal compression or neoplasia. Despite its neurosurgical relevance, comprehensive meta-analytic evidence synthesizing OMNP etiologies, clinical presentation, and contemporary diagnostic pathways remains limited. **Methods:** Following PRISMA 2020 guidelines, MEDLINE, Scopus, and Web of Science were systematically searched for studies reporting quantitative data on OMNP. Pooled prevalence estimates were calculated using random-effects models for causes and symptoms, while a structured narrative synthesis was performed for diagnostic modalities because outcome reporting was heterogeneous and unsuitable for meta-analysis. Risk of bias was assessed using the Joanna Briggs Institute (JBI) risk of bias tool. **Results:** Twenty-four studies involving 5541 patients were included. Using a multivariate multilevel model to account for within-study dependence, the most common etiological category was vascular disorders (35.62%), followed by idiopathic (16.47%) and neoplastic (12.10%). Head trauma and aneurysms accounted for 11.26% and 10.08% of cases, respectively. Diplopia (60.63%) and ptosis (54.12%) remained the predominant clinical symptoms, while pupil involvement was identified in 40.62% of the pooled population. Diagnostic paradigms have shifted decisively toward non-invasive neuroimaging, with magnetic resonance imaging reported in 66% of included studies and magnetic resonance or computed tomographic angiography increasingly employed to identify surgically relevant vascular lesions. **Conclusions:** Although vascular disorders represented the most common etiological category, the notable prevalence of aneurysmal and neoplastic causes underscores the importance of prompt high-resolution neuroimaging and early neurosurgical assessment. Early recognition and etiological stratification remain essential to optimize management and prevent irreversible neurological morbidity.

## 1. Introduction

Oculomotor nerve palsy (OMNP) results from dysfunction of the third cranial nerve (CN III) and is a common neuro-ophthalmological disorder affecting ocular motility and eyelid elevation. Complete loss of oculomotor nerve (OMN) function is defined as paralysis, whereas partial impairment is termed paresis [1,2]. Clinically, OMNP typically presents with a characteristic “down-and-out” ocular deviation due to unopposed action of the lateral rectus and superior oblique muscles, frequently accompanied by ptosis and, in some cases, pupillary dilation [3,4]. These findings reflect the selective involvement of the somatic motor and parasympathetic fibers of the OMN [5]. Early recognition is critical, as OMNP may represent the initial manifestation of a posterior communicating artery (PComA) aneurysm, a potentially life-threatening condition requiring urgent neurovascular evaluation [6]. The annual incidence of acquired OMNP is approximately 4.2 per 100,000, becoming increasingly common as age advances [3].

**Figure 1:**
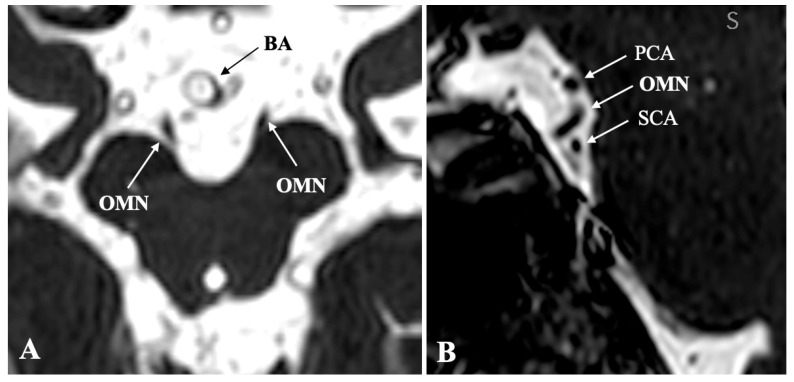
Anatomical relationships of the oculomotor nerve (OMN) in the interpeduncular cistern. (**A**) Axial magnetic resonance image demonstrating the bilateral OMN emerging from the midbrain and coursing anteriorly within the interpeduncular cistern, in close proximity to the basilar artery (BA). (**B**) Sagittal magnetic resonance image illustrating the OMN as it courses between the posterior cerebral artery (PCA) superiorly and the superior cerebellar artery (SCA) inferiorly. Image Credit: Dr. Nikolaos-Achilleas Arkoudis and Prof. Georgios Velonakis.

Anatomically, the OMN originates from a complex of subnuclei within the midbrain tegmentum at the level of the superior colliculus [7]. After exiting the midbrain through the interpeduncular fossa, the nerve traverses the subarachnoid space—characteristically passing between the posterior cerebral artery and the superior cerebellar artery—while advancing laterally and inferiorly to the posterior communicating artery (Figure 1). The nerve then passes through the lateral wall of the cavernous sinus before entering the orbit via the superior orbital fissure [7]. Within the orbit, it provides motor innervation to the levator palpebrae superioris, superior rectus, medial rectus, inferior rectus, and inferior oblique muscles. Its parasympathetic fibers synapse in the ciliary ganglion and subsequently innervate the sphincter pupillae and ciliary muscle, mediating pupillary constriction and lens accommodation [7].

Although several narrative reviews have addressed the etiologies, clinical features, and diagnostic evaluation of OMNP [1,2,8,9,10,11,12,13], these studies rely largely on descriptive summaries and lack quantitative pooled analyses. Consequently, the relative prevalence of etiological categories, symptom patterns, and diagnostic approaches remains unclear. The present study addresses this gap by providing a multilevel meta-analysis of OMNP etiologies and clinical manifestations, complemented by a structured narrative synthesis of diagnostic strategies.

## 2. Materials and Methods

### 2.1. Methodology

This systematic review was performed in accordance with the protocol proposed by the Preferred Reporting Items for Systematic Reviews (PRISMA) 2020 guidelines [14] (Supplementary Materials). According to the guidelines, the protocol was registered in the PROSPERO database with the number CRD420261302566. Risk of bias assessment for the studies was performed according to the Joanna Briggs Institute tool (JBI) [15]. Each study was evaluated for the presence of clearly defined inclusion criteria, valid measurement of the condition, consecutive and complete participant inclusion, adequate reporting of demographic characteristics and clinical information, appropriate follow-up or outcome reporting, clear description of the study setting, appropriate statistical analysis, and objective outcome assessment. Studies meeting seven or more of these criteria were classified as having low risk of bias, those meeting four to six criteria as moderate risk, and those meeting three or fewer criteria as high risk of bias [15].

### 2.2. Research Question

Following an extensive literature review, this study was designed to address the following research question: What are the etiologies and clinical manifestations of OMNP, and how is it diagnosed? Only studies reporting quantitative data specifically related to OMNP etiologies, symptoms, and diagnostic modalities were considered eligible. Studies involving concomitant cranial nerve palsies, ocular motility disorders not attributable to nerve palsy, case reports, narrative reviews, and conference abstracts were excluded.

### 2.3. Literature Search

Two independent reviewers (K.B., G.T.) performed the literature search and data extraction. The electronic databases MEDLINE (PubMed), Scopus, and Web of Science were used to search the primary literature until December 2025. The following keywords were used in several combinations: “oculomotor nerve”, “III nerve”, “third cranial nerve”, “palsy”, “paralysis”, “paresis”, “etiology”, “causes”, “clinical features”, “symptoms”, and “diagnosis”. No restrictions regarding publication date were applied. A secondary manual search of the reference lists of all eligible articles was also conducted to identify additional relevant studies. Data from the included studies were extracted into standardized Microsoft Excel spreadsheets prior to statistical analysis.

### 2.4. Statistical Meta-Analysis

Statistical analysis was conducted using the open-source R programming language and RStudio version 4.3.2, with the “meta” and “metafor” packages, by a single researcher (GT). To address the inherent within-study dependence of etiological and symptomatic proportions, where multiple outcomes originate from the same patient cohorts, a multivariate multilevel random-effects meta-analysis was performed. Data were modeled using a nested structure (etiologies/symptoms nested within studies) to accurately partition variance components. In contrast, diagnostic modalities were summarized descriptively through a narrative systematic synthesis, as quantitative pooling was not feasible because investigations were not mutually exclusive and reporting was inconsistent across studies. The proportions meta-analysis (prevalence meta-analysis) was conducted using the Freeman-Tukey double arcsine transformation, the DerSimonian-Laird estimator for the between-study variance tau^2^, and the Jackson method for the confidence interval of tau^2^ and tau. Cochran’s Q statistics were used to evaluate the presence of heterogeneity across studies, and the Higgins I^2^ statistic was used to quantify heterogeneity. Cochran’s Q *p*-value < 0.10 was considered significant. In the multivariate model, I^2^ was partitioned into Level 3 (between-study) and Level 2 (within-study/between-category) components to better explain the source of inconsistency. Higgins I^2^ values between 0 and 40% were regarded as low heterogeneity, 30–60% as moderate heterogeneity, 50–90% as substantial heterogeneity, and 75–100% as considerable heterogeneity. A *p*-value of less than 0.05 was considered statistically significant. To evaluate the presence of small-study effect (the phenomenon that smaller studies may show different effects than large ones), the DOI plot with the LFK index was generated [16].

## 3. Results

### 3.1. Selection Process

**Figure 2:**
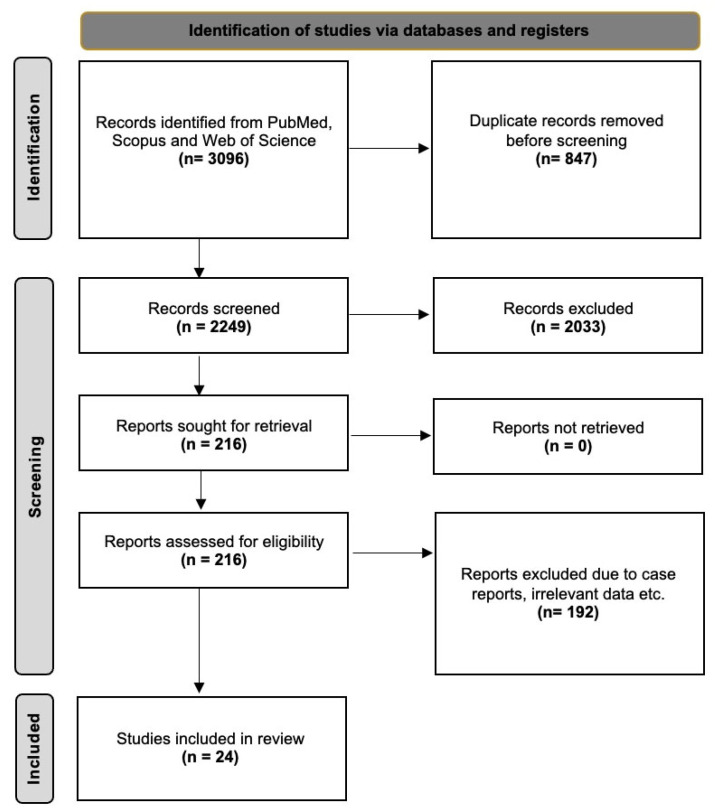
PRISMA 2020 flow diagram illustrating the study selection process.

The databases retrieved 3096 results that were exported to Mendeley version 2.10.9 (Elsevier, London, UK). After excluding irrelevant and duplicate papers, 216 studies were retrieved for full-text screening. Finally, 24 studies were included in the current systematic review. In accordance with the PRISMA 2020 guidelines [14], Figure 2 presents the flow diagram of the study selection process.

### 3.2. Studies’ Characteristics

The main demographic and methodological characteristics of the included studies are summarized in Table 1 and Table 2. Twenty-four studies comprising 5541 patients were included, with a mean sample size of 230.9 participants per study. The year of publication ranged from 1964 to 2024. Twelve studies were conducted in Asian populations, ten in American populations, and two in European populations. Age distribution, sex, and laterality are summarized in Table 2. Risk of bias assessment depicted 14 studies with low risk [17,18,19,20,21,22,23,24,25,26,27,28,29,30] and 10 studies with moderate risk [31,32,33,34,35,36,37,38,39,40].

### 3.3. Oculomotor Nerve Palsy Etiologies

To account for the inherent within-study dependence of etiological proportions, a multivariate random-effects meta-analysis was performed. The most frequent etiology of OMNP was vascular disorders, with a pooled prevalence of 35.62% (95% CI: 30.13–41.29). This was followed by unknown (idiopathic) causes at 16.47% (95% CI: 12.00–21.45) and the unified group of “other” causes (including post-neurosurgery, inflammation, and infections) at 15.45% (95% CI: 11.32–20.05). Neoplasms and head trauma accounted for 12.10% (95% CI: 8.36–16.36) and 11.26% (95% CI: 7.58–15.51) of cases, respectively, while aneurysms were identified in 10.08% of the pooled population (95% CI: 6.61–14.11).

Partitioned heterogeneity analysis revealed a total I^2^ of 93.66%. Within the multilevel model, the between-study heterogeneity component was minimal, although substantial overall heterogeneity persisted. However, the residual heterogeneity was entirely localized within the studies (93.65%), reflecting significant clinical and methodological diversity between etiological categories within each study.

**Figure 3:**
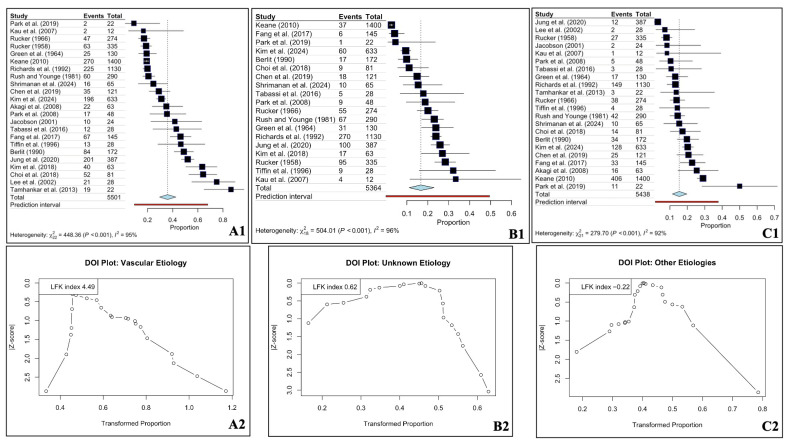
Forest (**A1**,**B1**,**C1**) and DOI (**A2**,**B2**,**C2**) plots for the oculomotor nerve palsy and the three most common etiologies—vascular (**A1**,**A2**), unknown (**B1**,**B2**), and other (**C1**,**C2**) [17,18,19,20,21,22,23,24,25,26,27,28,29,30,31,32,33,34,35,36,37,38,39,40].

The Forest and DOI plots for the three most common etiologies are present in Figure 3. The DOI plot of vascular etiology showed a marked asymmetry (Figure 3).

**Figure 4:**
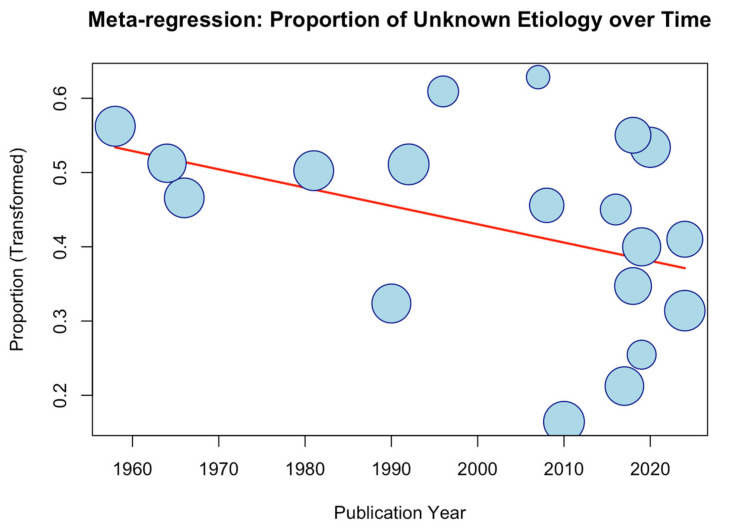
Meta-regression analysis of the oculomotor nerve palsy of unknown etiology based on year of publication.

A meta-regression analysis of idiopathic cases, stratified by publication year, showed a decreasing trend over time, although this did not reach statistical significance (*p* = 0.1213) (Figure 4).

### 3.4. Oculomotor Nerve Palsy Symptoms

Clinical symptoms were synthesized using a multivariate multilevel model to account for the reporting of multiple clinical features within the same study. The most common symptom was diplopia, with a pooled prevalence of 60.63% (95% CI: 30.93–86.71), followed by ptosis at 54.12% (95% CI: 36.39–71.35). Pain (periorbital or headache) was reported in 46.17% of cases (95% CI: 27.61–65.26), and pupil involvement was identified in 40.62% (95% CI: 26.06–56.03).

Additional clinical findings included partial paralysis [34.17% (95% CI: 11.37–61.53)], complete OMN dysfunction [25.03% (95% CI: 7.94–47.24)], and oculomotor synkinesis [11.11% (95% CI: 0.00–36.32)]. Heterogeneity analysis for the symptoms model yielded a total I^2^ of 97.37%. Similar to the etiological analysis, the majority of this variation was attributed to within-study differences between symptom categories (I^2^ = 88.65%), while the between-study component was low at 8.72%.

**Figure 5:**
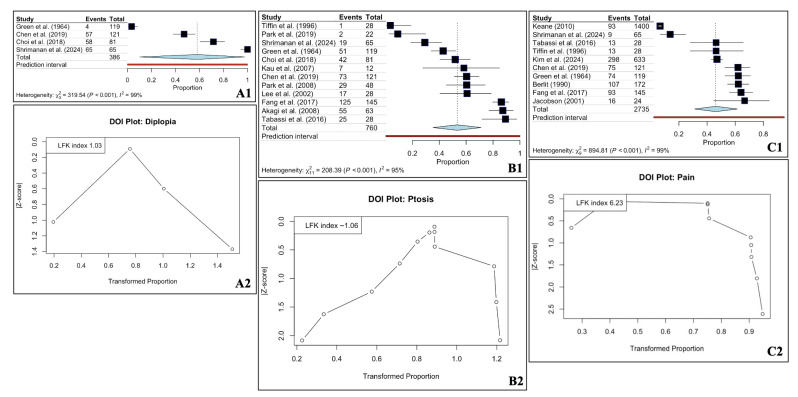
Forest (**A1**,**B1**,**C1**) and DOI (**A2**,**B2**,**C2**) plots for the oculomotor nerve palsy and the three most common symptoms—diplopia (**A1**,**A2**), ptosis (**B1**,**B2**), and pain (**C1**,**C2**) [17,18,19,20,21,22,23,24,25,26,27,28,29,30,31,32,33,34,35,36,37,38,39,40].

The Forest and DOI plots for the three most common symptoms are present in Figure 5. All the DOI plots exhibited asymmetry (Figure 5). Given the wide confidence intervals of several pooled estimates, these prevalences should be interpreted as descriptive summaries of reported cohorts rather than predictive clinical probabilities.

### 3.5. Narrative Synthesis of Diagnostic Modalities in Oculomotor Nerve Palsy (Table 3)

Diagnostic investigations were frequently non-mutually exclusive, with several studies reporting combinations of imaging and invasive modalities within the same cohorts. Therefore, these findings are presented descriptively rather than synthesized through quantitative meta-analysis. Magnetic resonance imaging (MRI) was the most commonly utilized modality, reported in 16 of the 24 studies, and was often complemented by magnetic resonance angiography (MRA) in seven more recent cohorts.

**Table 3:** Diagnostic modalities used across the studies to identify oculomotor nerve palsy. MRI = magnetic resonance imaging; MRA = magnetic resonance angiography; CT = computed tomography; CTA = computed tomographic angiography; LP = lumbar puncture; DSA = digital subtraction angiography; NR = not reported.

Author (Year)	MRI	MRA	CT	CTA	LP	DSA	X-Ray
Akagi et al. [17]	X	X	X				
Berlit [38]			X		X	X	X
Chen et al. [18]	X	X	X	X	X	X	
Choi et al. [27]	X	X			X		
Fang et al. [19]	X	X	X				
Green et al. [37]					X	X	X
Jacobson [33]	X	X	X			X	
Jung et al. [20]					X		
Kau et al. [32]	X						
Keane [21]	X		X		X		
Kim et al. [22]	X						
Kim et al. [23]	X	X					
Lee et al. [24]	X				X	X	
Park et al. [25]	NR						
Park et al. [30]	X						
Richards et al. [35]	X		X			X	
Rucker [31]						X	
Rucker [32]	NR						
Rush et al. [36]			X				
Srimanan et al. [26]	NR						
Tabassi et al. [28]	X	X					
Tamhankar et al. [29]	X		X				
Tiffin et al. [39]	X		X			X	
Zhang and Wei [40]	X		X			X	

Computed tomography (CT) remained a consistent diagnostic tool, utilized in 11 studies ranging from early retrospective reviews to contemporary clinical analyses.

Invasive procedures such as digital subtraction angiography (DSA) and lumbar puncture (LP) were employed in 9 and 7 studies, respectively, particularly when identifying aneurysms or inflammatory causes.

Computed tomographic angiography (CTA) was only explicitly reported as a modality in the study by Chen et al. [18].

Conventional X-ray was largely restricted to older literature, appearing only in studies by Berlit [38] and Green et al. [37].

Diagnostic modalities were not reported (NR) in three of the evaluated studies [21,22,30].

## 4. Discussion

**Figure 6:**
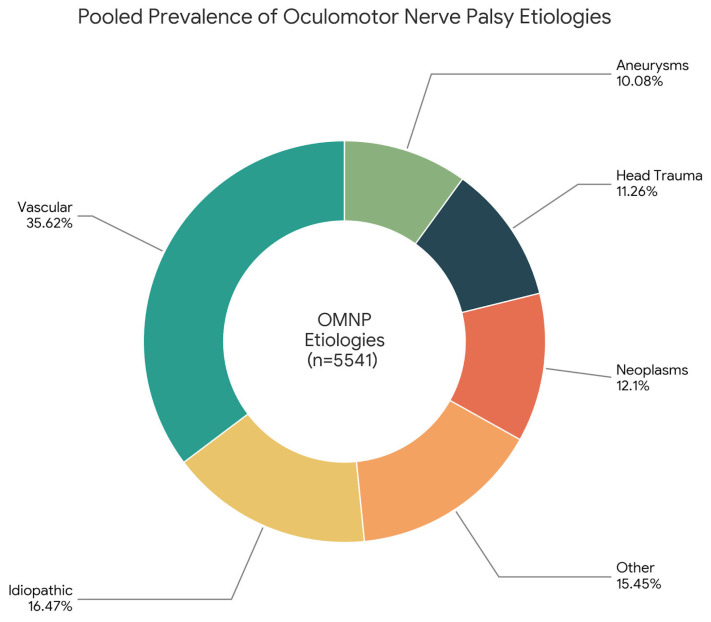
Graphical representation of the oculomotor nerve palsy etiologies based on their pooled prevalence.

This systematic review and meta-analysis provide the most comprehensive quantitative synthesis to date of the etiologies, clinical manifestations, diagnostic strategies, and treatment considerations of OMNP. By integrating data from 24 studies spanning more than six decades, several clinically meaningful insights emerge. Most notably, vascular disorders (with microvascular ischemia as the leading cause) were the predominant cause of acquired OMNP across most cohorts (Figure 6).

**Figure 7:**
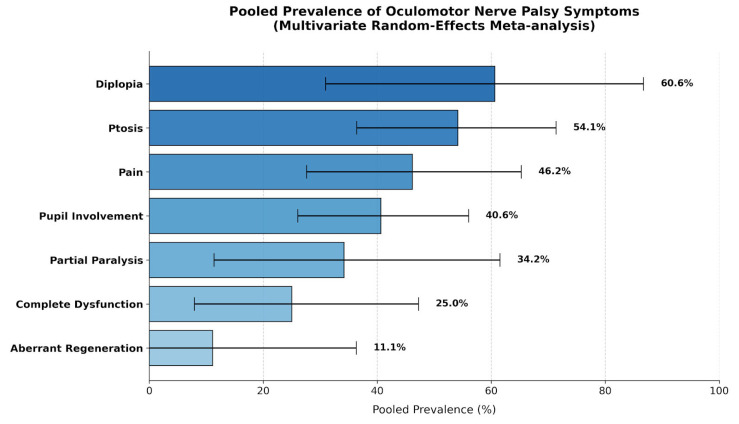
Graphical representation of the oculomotor nerve palsy symptoms based on their pooled prevalence.

Binocular diplopia and ptosis emerged as the most consistent clinical manifestations, irrespective of etiology, occurring in a substantial proportion of patients (Figure 7). Concurrently, the diagnostic approach to OMNP has evolved substantially, with a clear and sustained shift toward non-invasive neuroimaging modalities.

### 4.1. Oculomotor Nerve Palsy Etiologies

#### 4.1.1. Vascular Etiologies

The findings of the present systematic review and meta-analysis confirm that vascular disorders represent the most frequent etiological category of OMNP. This group is predominantly driven by microvascular ischemia, which is strongly associated with systemic risk factors such as hypertension, diabetes mellitus, and atherosclerosis [29,36]. Diabetes mellitus, in particular, has traditionally been associated with pupil-sparing clinical presentations, reflecting preferential ischemic involvement of peripheral OMN fibers. However, advances in neuroimaging have expanded this paradigm, with focal midbrain infarctions now recognized as a central mechanism of OMNP in selected vasculopathic patients [41].

**Figure 8:**
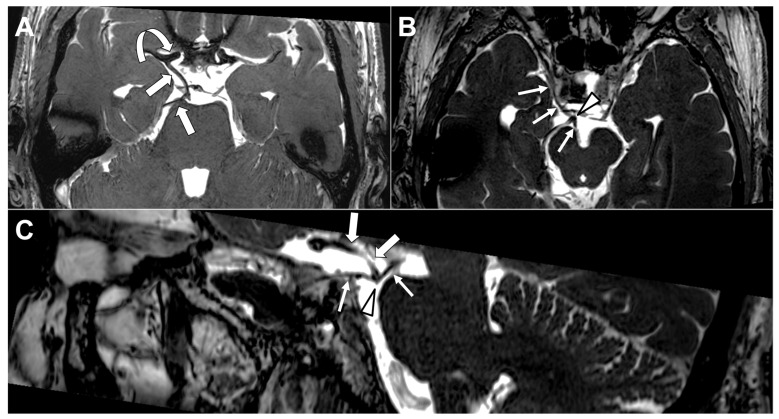
Fetal-origin posterior cerebral artery (PCA) causing neurovascular conflict with the oculomotor nerve (OMN). (**A**) Axial 3D high-resolution T2-weighted balanced fast field echo (BFFE) image with minimum intensity projection (MinIP) reconstruction demonstrates a fetal-origin right PCA (thick straight arrows) arising directly from the supraclinoid segment of the internal carotid artery (curved thick arrow). (**B**) A different MinIP reconstruction shows focal malalignment and “tenting” of the cisternal segment of the right OMN (thin arrows) at the site of vascular contact (arrowhead), consistent with compressive neurovascular conflict by the fetal PCA. (**C**) Sagittal MinIP reconstruction clearly depicts the angulation and inferior displacement of the cisternal OMN (thin arrows) produced by the adjacent fetal-origin PCA (thick arrows) at the site of neurovascular conflict (arrowhead). Image Credit: Dr. Nikolaos-Achilleas Arkoudis and Prof. Georgios Velonakis.

Another vascular mechanism involves mechanical compression of the OMN by aberrant arterial structures. Owing to its course between the posterior cerebral artery and the superior cerebellar artery, the OMN is particularly susceptible to neurovascular contact [40,42]. However, the radiological contact that can be frequently observed does not always translate into clinical symptoms [40,42]. Symptomatic neurovascular compression syndromes typically arise when such contact involves the transition zone between central and peripheral myelin [41,43]. Several isolated cases were reported where aberrant arteries clinically compressed the OMN [42,44,45]. Figure 8 shows a fetal-origin posterior cerebral artery causing neurovascular conflict with the OMN.

The susceptibility of the OMN to ischemic and compressive injury may partly reflect its complex microanatomy, vascular supply, and segment-specific fiber organization. Emerging histological evidence from embalmed donor tissues suggests that preserved neural specimens can still provide valuable insights into the microstructure of the central and peripheral nervous systems, supporting the continued relevance of cadaveric anatomical studies for understanding cranial nerve vulnerability and age-related pathological changes [5].

#### 4.1.2. Uncommon Etiologies

**Figure 9:**
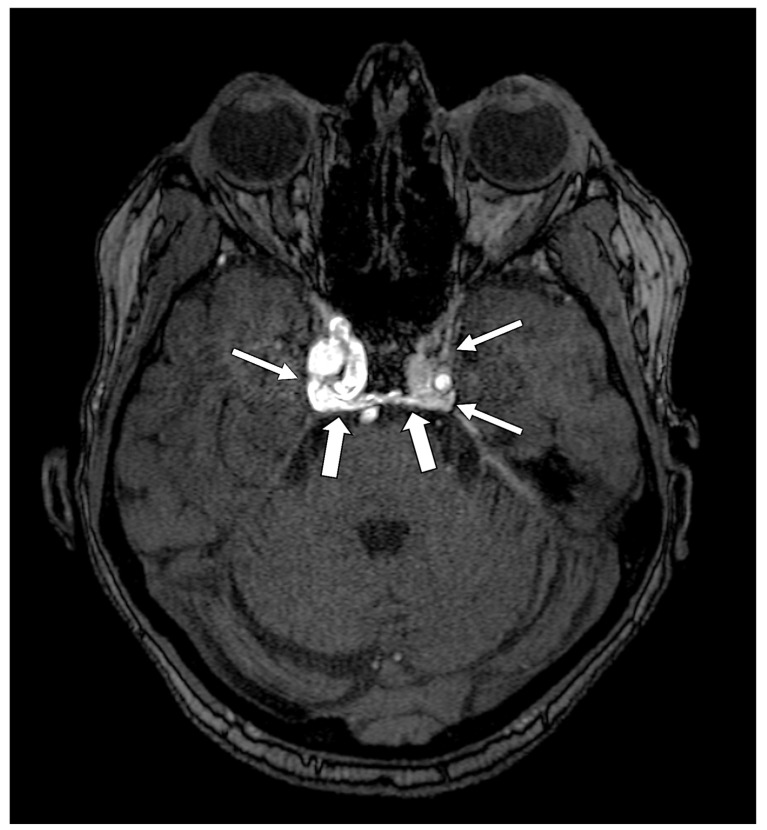
Time-of-Flight (TOF) MR Angiography in a Patient with Carotid Cavernous Fistula (CCF). Axial three-dimensional Time-of-Flight (TOF) MR angiography demonstrates an abnormally high-flow signal bilaterally within the cavernous sinus (thin arrows) with asymmetric enlargement most prominent on the right side, findings consistent with a CCF. Also note that there is early venous filling extending in the dilated basilar venous plexus and petrosal sinus (thick arrows), further supporting the presence of high-flow arteriovenous shunting. Clinically, the patient had presented with progressive orbital symptoms, including pulsatile proptosis, conjunctival chemosis, diplopia, and oculomotor nerve (OMN) palsy (ptosis and impaired extraocular movements). These imaging findings correlate with the patient’s OMN dysfunction due to compression and vascular congestion within the cavernous sinus. Image Credit: Dr. Nikolaos-Achilleas Arkoudis and Prof. Georgios Velonakis.

**Figure 10:**
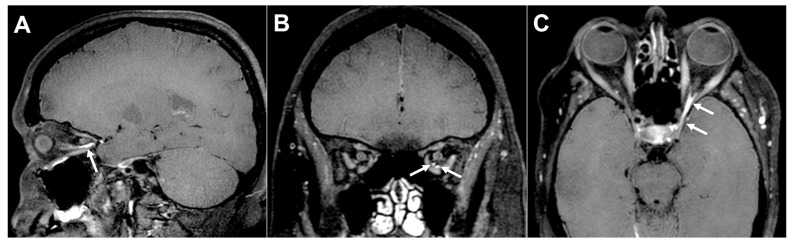
Post-contrast black-blood MRI demonstrating enhancement of the left oculomotor nerve (OMN) consistent with inflammatory neuropathy. (**A**) Sagittal, (**B**) coronal, and (**C**) axial post-contrast black-blood T1-weighted images demonstrate smooth, linear enhancement with only mild thickening of the cavernous segment of the left OMN, extending anteriorly into its orbital division through the superior orbital fissure (arrows). The enhancement is continuous along the expected anatomic course of the nerve, without nodularity or a discrete mass. The adjacent cavernous sinus is not expanded, and there is no evidence of vascular abnormality or compressive lesion. The imaging findings support inflammatory oculomotor neuritis as the underlying cause. The patient had presented with diplopia and ptosis. Image Credit: Dr. Nikolaos-Achilleas Arkoudis and Prof. Georgios Velonakis.

**Figure 11:**
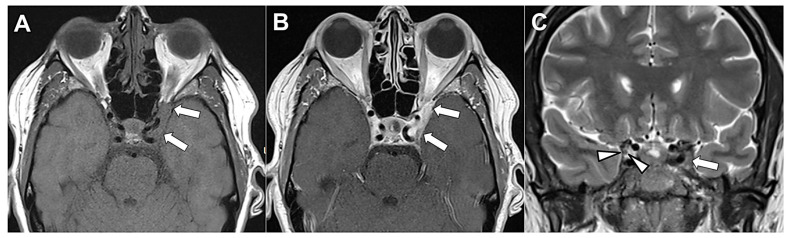
MRI findings in left-sided Tolosa–Hunt syndrome with oculomotor nerve (OMN) involvement. (**A**) Axial T1-weighted non-fat-saturated image demonstrates hypointense soft tissue replacing and mildly expanding the left cavernous sinus, extending toward the superior orbital fissure (thick arrows). (**B**) Axial T1-weighted post-contrast non-fat-saturated image shows homogeneous abnormal enhancement of this soft tissue (thick arrows). (**C**) Coronal T2-weighted image shows the lesion as mildly hypointense, with associated mild expansion of the left cavernous sinus (thick arrow). Note how the right OMN is clearly visualized within the right normal cavernous sinus (arrowheads), whereas the left OMN cannot be seen due to the surrounding abnormal tissue. Clinically, the patient had presented with acute unilateral periorbital pain, ipsilateral ptosis, diplopia, and impaired adduction, elevation, and depression of the left eye. Therefore, overall, the imaging findings were consistent with Tolosa–Hunt syndrome, an idiopathic granulomatous inflammatory process involving the cavernous sinus and causing OMN palsy secondary to cavernous sinus inflammation. Image Credit: Dr. Nikolaos-Achilleas Arkoudis and Prof. Georgios Velonakis.

The “other” etiological category encompasses a heterogeneous group of less common but clinically relevant conditions. This classification reflects a heterogeneous group of conditions that includes post-neurosurgery [21], congenital OMNP [35], and vascular anomalies distinct from simple ischemia, such as carotid cavernous fistulae (Figure 9) [18]. Inflammatory conditions (Figure 10), mainly multiple sclerosis [34] and Guillain-Barré syndrome [20], are also prominent in this group. Furthermore, various infections and infectious syndromes are included, such as syphilis [37], herpes zoster ophthalmicus [36], and Tolosa-Hunt syndrome (Figure 11) [21,33].

#### 4.1.3. Aneurysmal Etiologies

**Figure 12:**
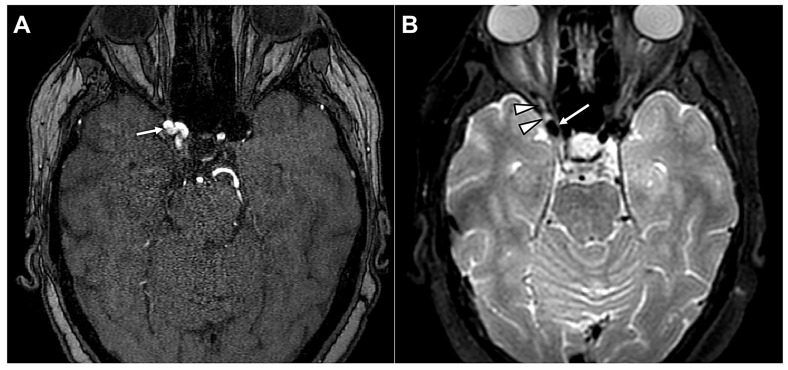
MRI findings of a supraclinoid internal carotid artery (ICA) aneurysm with secondary oculomotor nerve (OMN) compression. (**A**) Axial Time-of-Flight (TOF) MR angiography demonstrates a saccular aneurysm arising from the supraclinoid segment of the ICA, projecting laterally (arrow). (**B**) Corresponding axial T2-weighted imaging reveals thickening and hyperintensity of the adjacent OMN (arrowheads). The findings were compatible with edema secondary to mass effect from compressive neuropathy due to the aneurysm (arrow). The patient’s symptoms included third nerve palsy and pupillary dilation. Image Credit: Dr. Nikolaos-Achilleas Arkoudis and Prof. Georgios Velonakis.

Aneurysms represent a clinically critical etiology of OMNP, given their risk of rupture and subsequent subarachnoid hemorrhage [11]. OMNP caused by an aneurysm is more often related to the PComA [46], specifically at the junction of the internal carotid artery (ICA) [12]. Aneurysms of the supraclinoid segment of the ICA are also well-recognized causes of third cranial nerve dysfunction (Figure 12) [21]. Other identified locations include the terminal segments of the ICA and the basilar artery [11]. Given their acute neurosurgical significance, aneurysmal etiologies are appropriately prioritized in contemporary diagnostic algorithms for OMNP.

#### 4.1.4. Neoplastic Etiologies

**Figure 13:**
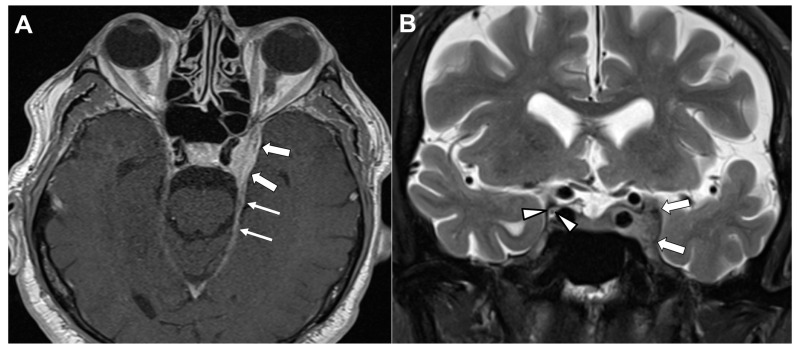
MRI features of left cavernous sinus meningioma with oculomotor nerve (OMN) involvement. (**A**) Axial T1-weighted post-contrast (non-fat-saturated) image demonstrates a homogeneously enhancing soft tissue lesion filling and expanding the left cavernous sinus (thick arrows), encasing the cavernous segment of the internal carotid artery without significant luminal narrowing. Contrast enhancement extends posteriorly along the tentorium (thin arrows), a finding known as the “dural tail sign”, supporting a dural-based neoplasm. (**B**) Coronal T2-weighted fat-saturated image shows the lesion to be iso- to mildly hypointense relative to gray matter, with mass effect upon the lateral wall of the cavernous sinus and obscuration of the expected course of the OMN. Note how the right OMN is clearly visualized within the right normal cavernous sinus (arrowheads), whereas the left OMN cannot be distinguished due to its compression (arrows). The imaging features are characteristic of a cavernous sinus meningioma. The patient had presented with progressive ptosis, diplopia, and impaired extraocular movements. Image Credit: Dr. Nikolaos-Achilleas Arkoudis and Prof. Georgios Velonakis.

Neoplasms represent a critical etiology for OMNP, as dysfunction of the OMN can often serve as the earliest clinical sign of a brain tumor [17]. The most common neoplasms identified as causes include meningiomas (Figure 13) [23] and pituitary adenomas, which are frequently apoplectic and metastatic lesions from solid tumors [21]. Additional neoplastic etiologies documented in the literature include gliomas, nasopharyngeal carcinomas [25], craniopharyngiomas, and nerve-specific lesions such as oculomotor nerve schwannomas [21]. Tumor-related OMNP most commonly results from direct mechanical compression, perineural infiltration, or invasion of adjacent structures, particularly the cavernous sinus [21].

#### 4.1.5. Idiopathic Etiologies

Despite comprehensive diagnostic evaluations, OMNP remains of unknown or undetermined origin in 16.47% of patients. While it was historically anticipated that advancements in imaging would eliminate these idiopathic cases, longitudinal data suggest that several cases still lack a definitive causal diagnosis at initial evaluation. This finding is supported by the present meta-regression analysis, which demonstrated no statistically significant temporal decline in the prevalence of idiopathic OMNP.

### 4.2. Oculomotor Nerve Palsy Symptoms

While our meta-analysis provides pooled prevalence estimates for major symptoms, these figures should be interpreted as descriptive benchmarks rather than predictive diagnostic values, given the substantial statistical uncertainty reflected in the wide confidence intervals. Based on these, diplopia emerged as the most frequently reported symptom of OMNP and often represented the primary reason for clinical presentation, particularly in cases of isolated nerve involvement. Ptosis was the second most common symptom and could be either partial or complete [37].

Pupil involvement was identified in 40.62% of the pooled population. In the context of this study, this term refers to anisocoria (unequal pupil size) or a nonresponsive pupil (fixed pupil) [33]. The pupil’s status remains a vital diagnostic differentiator. Although complete pupil sparing is traditionally associated with microvascular ischemia, even partial pupillary dysfunction is a high-risk indicator for compressive lesions, such as posterior communicating artery aneurysms or neoplasms [28,38].

Pain was reported in 46.17% of patients. This symptom describes unilateral periorbital pain or headache ipsilateral to the nerve palsy. While intense pain is often considered associated with aneurysmal expansion or rupture, our findings and supporting literature indicate that pain is also remarkably common in ischemic cases or diabetic or hypertensive OMNP [10,21].

The degree of OMN dysfunction varied, with partial paralysis noted in 34.17% of cases and complete dysfunction in 25.03%. Distinguishing between these states is clinically significant, as complete external ophthalmoplegia with pupillary sparing strongly suggests a non-compressive vascular cause [24].

Finally, oculomotor synkinesis, also referred to as aberrant regeneration, was the least common finding. This phenomenon, characterized by misdirected nerve fibers causing unintended eye or lid movements, is typically seen in traumatic or chronic compression etiologies and is almost never observed in primary microvascular cases [19,32].

### 4.3. Oculomotor Nerve Palsy Diagnosis

The diagnostic framework for OMNP has transitioned from early reliance on clinical observation and invasive testing towards a highly refined neuroimaging approach. MRI was the most frequently reported modality across the 24 studies, utilized as a primary tool to differentiate between structural lesions and ischemic events. In more recent cohorts, the integration of MRA has further enhanced the detection of intracranial aneurysms, significantly reducing the immediate need for catheter-based procedures. MRI integration was the most frequently reported modality and is currently central to OMNP evaluation. In recent cohorts, combined MRI/MRA improved identification of structural and vascular causes while reducing reliance on invasive angiography [12,27,47]. Formal pooled sensitivity and specificity estimates could not be generated because the included studies were predominantly retrospective observational cohorts rather than diagnostic accuracy studies using standardized reference standards.

CT with or without CTA remained critical in acute settings, particularly for screening patients for subarachnoid hemorrhage or large aneurysms, since the smallest aneurysms causing OMNP are found to be at least 4 mm, namely within the range of detection of these techniques [13,18]. Even though invasive modalities such as DSA and LP were fundamental in older literature, they are at present reserved mostly for cases where non-invasive imaging is inconclusive or when an inflammatory or infectious cause is suspected [18,40]. This structured combination of modalities ensures the prompt identification of neurosurgical emergencies while refining the diagnostic pathway for idiopathic or microvascular cases.

### 4.4. Oculomotor Nerve Palsy Treatment Options

The management of oculomotor nerve palsy is inherently tied to its etiology, ranging from conservative observation in microvascular cases to urgent surgical or endovascular intervention for aneurysmal compression. For OMNP induced by posterior communicating artery aneurysms, the two primary interventions are microsurgical clipping and endovascular treatment, such as coiling [48,49]. Meta-analytic evidence indicates that microsurgery is associated with a significantly higher likelihood of favorable OMNP recovery compared with endovascular treatment at 1, 3, 6, and 12 months [49]. Specifically, early recovery rates are more robust with microsurgery, showing 53% favorable recovery at 1 month compared to only 17% for endovascular intervention, likely because surgery promptly removes the aneurysmal mass effect and ceases repetitive arterial pulsations against the nerve [49]. The duration of symptoms prior to treatment is a critical predictor of functional recovery. Patients receiving intervention within an ‘early window’ (7–14 days) have significantly higher odds of achieving complete recovery compared to those treated later [49]. When OMNP is chronic or results from trauma where the nerve does not spontaneously recover, strabismus surgery is the treatment. The primary goal is to achieve gaze alignment in the primary position and resolve ptosis to improve aesthetics and quality of life [50]. Despite these advancements, post-operative recurrence is a documented challenge. Exotropic drift frequently occurs as the denervated medial rectus is eventually overcome by the tone of the lateral rectus, often necessitating intentional overcorrection of 5–15 degrees [50]. Furthermore, ptosis correction via frontalis suspension has a documented 12-month recurrence rate of approximately 26% [50]. Evidence regarding structured rehabilitation strategies, including orthoptic therapy or formal physiotherapy-based ocular motor retraining, was scarce and inconsistently reported across the included literature.

### 4.5. Strengths and Limitations

The current systematic review and meta-analysis address a significant gap in the literature by providing comprehensive meta-analytic evidence regarding the etiologies, symptoms, and diagnostic modalities of OMNP. The study includes a robust total sample of over 5000 patients from 24 studies, providing high statistical power for prevalence estimates. Methodologically, by employing an advanced multivariate multilevel statistical framework, this study accounts for the correlation between clinical variables within the same study cohorts—a nuance often overlooked in previous reviews. This enabled more precise estimation of confidence intervals and clearer partitioning of heterogeneity. Additionally, several included studies spanned earlier decades, during which neuroimaging resolution, diagnostic criteria, and etiological classification systems differed substantially from contemporary standards.

Despite these strengths, several limitations must be acknowledged. Primarily, a methodological challenge we encountered was the difficulty in extracting isolated quantitative data for OMNP from the existing literature. A significant number of the analyzed studies reported etiologies and symptoms for a combined cohort of patients presenting with various acquired ocular motor nerve palsies, including those involving the trochlear and abducens nerves. Therefore, an important number of studies (10 out of 24) were classified as ‘moderate’ risk of bias according to the JBI tool. A significant limitation regarding clinical interpretability is the wide confidence intervals associated with several pooled symptom estimates. These intervals reflect the high Level 2 heterogeneity (88.65%) observed within our model, likely stemming from differences in how symptoms were documented and the retrospective nature of the largest included cohorts. The total heterogeneity remained high, exceeding 90% in several models. While our multilevel analysis demonstrated that the vast majority of this variation was localized within studies (Level 2) rather than between them (Level 3), it is critical to distinguish this statistical partitioning from true clinical homogeneity. The high Level 2 variance is likely driven by substantial differences in diagnostic criteria, etiological classification systems, and the evolution of neuroimaging resolution. These factors ensure that while the broad statistical trend is consistent, individual study outcomes remain influenced by their specific clinical and temporal contexts. Finally, the presence of major asymmetry in specific DOI plots suggests a potential small-study effect, particularly in rarer etiological groups where smaller cohorts may overrepresent specific outcomes.

Future studies should transition toward prospective multicenter designs with a standardized diagnostic and reporting framework, anatomical investigation of the topographical relationship, and long-term follow-up of the patients.

## 5. Conclusions

This study provides a comprehensive meta-analytic synthesis of OMNP etiologies and symptoms alongside a systematic narrative review of diagnostic trends in 5541 patients. Vascular disorders were the most prevalent etiological category, while aneurysmal and neoplastic causes remain high-risk entities with direct neurosurgical implications that require urgent exclusion. Diplopia and ptosis were the most common clinical manifestations, whereas pupillary involvement remains an important warning sign of compressive pathology. These pooled estimates should be interpreted as descriptive summaries of reported cohorts. Diagnostic pathways have progressively shifted toward non-invasive imaging, with MRI, often combined with MRA, representing the current first-line strategy in many centers. Future prospective multicenter studies using standardized diagnostic criteria are warranted.

## Figures and Tables

**Table 1:** Characteristics of the included studies with risk of bias assessment based on the Joanna Briggs Institute (JBI) Critical Appraisal Checklist for Prevalence Studies.

Author	Year	Country	Type	Sample Size	Risk of Bias
Akagi et al. [17]	2008	Japan	Retrospective Study	63	Low
Berlit [38]	1990	Germany	Retrospective Study	172	Moderate
Chen et al. [18]	2019	China	Retrospective Study	121	Low
Choi et al. [27]	2018	Republic of Korea	Prospective Multicenter Study	81	Low
Fang et al. [19]	2017	USA	Retrospective Study	145	Low
Green et al. [37]	1964	USA	Retrospective Study	130	Moderate
Jacobson [33]	2001	USA	Retrospective Study	24	Moderate
Jung et al. [20]	2020	Republic of Korea	Retrospective Study	387	Low
Kau et al. [32]	2007	USA	Prospective Case Series	12	Moderate
Keane [21]	2010	USA	Retrospective Study	1400	Low
Kim et al. [22]	2018	Republic of Korea	Retrospective Study	63	Low
Kim et al. [23]	2024	Republic of Korea	Retrospective Study	633	Low
Lee et al. [24]	2002	Republic of Korea	Retrospective Study	28	Low
Park et al. [25]	2008	Republic of Korea	Retrospective Study	48	Low
Park et al. [30]	2019	Republic of Korea	Retrospective Study	22	Low
Richards et al. [35]	1992	USA	Retrospective Study	1130	Moderate
Rucker [31]	1958	USA	Retrospective Study	335	Moderate
Rucker [34]	1966	USA	Retrospective Study	274	Moderate
Rush et al. [36]	1981	USA	Retrospective Study	290	Moderate
Srimanan et al. [26]	2024	Thailand	Retrospective Study	65	Low
Tabassi et al. [28]	2016	Iran	Retrospective Study	28	Low
Tamhankar et al. [29]	2013	USA	Prospective Multicenter Study	22	Low
Tiffin et al. [39]	1996	UK	Retrospective Study	28	Moderate
Zhang and Wei [40]	2019	China	Retrospective Study	40	Moderate

**Table 2:** Demographic characteristics of the included studies. Data were not available for each cohort; therefore, this table does not reflect the analysis of all the patients included in the systematic review. Demographic data were incompletely reported across studies and pooled descriptively.

Age	Mean (SD)	
	53.55 years old (11.43)	
Sex	Female (%)	Male (%)
	1460 (45.4%)	1750 (54.6%)
Laterality	Unilateral (%)	Bilateral (%)
	3870 (95.5%)	182 (4.5%)
Sample Size	Mean (Range)	
	230.9 (12–1400)	

## Data Availability

No new data were created or analyzed in this study. Data sharing is not applicable to this article.

## Supplementary Materials

The following supporting information can be downloaded at: https://www.mdpi.com/article/10.3390/diagnostics16091401/s1, PRISMA 2020 Checklist.
